# An Observational Study on Clothing Characteristics Involved as Major Contributors in Sustaining Domestic Burns Injuries

**DOI:** 10.29252/wjps.8.3.293

**Published:** 2019-09

**Authors:** Honnegowda Thiitamaranahalli Muguregowda

**Affiliations:** Department of Plastic Surgery and Burns, Kasturba Medical College, Manipal University. Udupi, India

**Keywords:** Burn, Injury, Garment, Clothing, India

## Abstract

**BACKGROUND:**

Fire and burn-related injuries are a leading cause of morbidity and mortality worldwide, and is a serious public health problem in developing countries. Several studies showed causes such as low socioeconomic status, poor living conditions, illiteracy, and floor level cooking, however, very few studies stated severity of the burn injuries to be dependent on ignition of type clothing garment and fabric wore at the time of incident.

**METHODS:**

A cross sectional observational study done on burn injury patients admitted from February 2014 to August 2016. Data were collected from the patients or their relatives and analysed.

**RESULTS:**

Among 224 burn injury patients, majority were females (59.3%) sustained burn injuries in the study population (*p*=0.005). Victims wearing long loose flowing garments such as sarees (41.1%), salwar (22.3%), and dupatta (9.8%) were caught fire easily and sustained more burn injuries, compared to clothes reaching down to the knee and short fitting dresses (*p*=0.004). Percentage of burn was higher among wearers of synthetic fabrics (50.89%) than that of cottons (20.53%, *p*=0.028].

**CONCLUSION:**

Every year, thousands of people are injured when their clothing catches fire. The findings reported herein documented that public knowledge about clothing related fire risks was lacking. This can be reduced by bringing about stronger regulations by government and to educate about the magnitude of the problems inflicted by burn injuries and to oversight and to promote less inflammable fabrics to be worn at home, especially in kitchen.

## INTRODUCTION

Injuries are increasingly and are recognized as a public health problem. Burns account for 1% of the global burden of diseases,^1^ cause more than 7.1 millions injuries, the loss of almost 18 million disability-adjusted life years (DALYs), and more than 265,000 deaths worldwide annually.^2^ Moreover, burns are ranked 4^th^ among all injuries causing not only deaths, but also major economic and psychological impacts and long-term somatic sequelae as well.^3^ The major factors associated with burn injuries are low socioeconomic status, poor living conditions, illiteracy, overcrowding and floor level cooking are risk factors frequently associated with burns.^4^^,^^5^

Study by Sanghavi *et al.* showed that average ratio of fire-related deaths of young women to men was 3:1.^6^ This could be attributed to the male dominant society and females′ close proximity to fire throughout the day and night. Parray *et al.* reported that in India, age group of 21–40 years were more prone to higher percentage of burns and mortality with providing reasons such as: women were susceptible because of household and kitchen responsibility, dowry harassment and by the nature of clothing worn by women in India such as saree and dupatta and thereby, practices of females in most households regarding their clothing attire worn over heads (dupatta) were also an important factor in increasing burn injuries.^7^

Since one-third of the clothing fire accidents initiates in the kitchen, females are involved in almost 65% (fatal and non-fatal) fire accidents.^8^ The main reason for higher levels of severe fatal accidents in the high-risk female group, is that because of the potentially loose fitting/flowing garments types associated with them. Literature suggests that approximately 40% of the dwelling fires happen in the kitchen, with an added probability of females wearing loose fitting garments, the chances of them getting involved in a clothing related fire is significantly high.^9^

It has also been observed that burns involving the ignition of clothing (loose fitting garments in particular) usually prove to be more severe because of the intimate nature of the clothing textiles. Feller *et al.* concluded in their study that in patients, burns associated with clothing ignition had a fourfold increase in mortality and a prolonged hospital stay (21 days longer) as compared to those patients whose clothing was not burned.^10^ Bhalla *et al.* in their study regarding burn characteristics of fabric used in India reported that loose fittings garments, nightgowns, kurta, etc. bum vigorously and with large flames, whereas tight fitting garments were difficult to burn.^11^

Dense fabrics such as ‘khadi,’ burn slower than thin cotton saree. However as there is no detailed studies available in recent literature, the present study was designed to identify how various cloth materials in household female at the time cooking were responsible for burn injuries. Further, an effort was made to correlate it with socioeconomic status and to recommend various preventive measures at the community level to decrease the incident of burn injury. 

## MATERIALS AND METHODS

This is an observational study conducted on total of 224 burn patients admitted between the periods February 2014 and August 2016, Burn Unit of Kasturba Medical College and Hospital, Manipal, India which is the only referral centre for major burns around neighbouring districts of Karnataka. Prospective demographic data regarding patients age, gender, mode and cause of burn, total burn surface area (TBSA), place of burn, material of clothing worn by the victim at the time of the incident, socio-economic status were collected after informed consent was provided from the patients themselves if they were able enough or from their attendants on a pre-designed questionnaire. 

The study protocol and informed consent form were reviewed and approved by Institutional Ethics committee, Kasturba Hospital, Manipal, India. The distribution of the data was checked for normality using Kolmogorov-Smirnov test. Since the data was not normally distributed non-parametric tests were carried out using percentages and Chi-Square test. Two-sided P-values were considered statistically significant at *p*<0.05. Results were expressed as mean±standard deviation (SD) unless otherwise indicated. All data were analysed using SPSS software (Version 22, IBM SPSS Inc., Chicago, IL, USA). 

## RESULTS

Out of 224 burn patients admitted during the study period, 133 (59.3%) were females. The male to female ratio was 0.8:1. The age range was from 10 to 70 years. Majority (55.8%) of patients were between 21 and 40 years of age followed by 33.9% that were less than 20 years of age (Table 1). The majority of patients (75.25%) were hailing from rural areas and 55.5% of the injured were from nuclear families. Maximum number (63.31 %) was married at the time of injury and 32.59% were housewives followed by 24.45% unskilled workers. It was observed that the majority of injuries occurred between 8 pm and 12 am. At the time of injury, 48.75% of victims were wearing synthetic clothes (*p*=0.001, Table 2). 

**Table 1:** Age distribution according to gender

**Age (years)**	**Male (%)**	**Female (%)**	**Total (%)**
<20	25 (11.16)	46 (20.5)	76 (33.9)
21-40	55 (24.55)	70 (31.25)	125 (55.8)
41-60	10 (4.46)	15 (6.69)	25 (11.16)
61-80	1 (0.44)	2 (0.89)	3 (1.33)
Total	91 (40.6)	133 (59.3)	224 (100)

(Chi square test) X^2^=3.58, df=1, *p*=0.005

**Table 2:** Distribution according to material of clothing worn by male and female at the time of burn

**Material of clothing**	**Male (%) **	**Female (%) **	**Total (%)**
Cotton	30 (31.13)	16 (8.2)	46 (18.34)
Semi-synthetic	43 (49.06)	22 (16.42)	65 (30.83)
Synthetic	22 (16.04)	91 (74.63)	113 (48.75)

(Chi square test) X^2^=10.66 DF=2, *p*=0.004

The majority of the victims were females (59.3%) and suffered from burns more than 50% TBSA, whereas males (42.41%) suffered from burns nearly 20% TBSA (*p*=0.004, Table 2). All patients sustained burns because their clothes caught fire. Victims wearing the following long loose flowing garments such as sarees (41.1%), salwar (22.3%), and dupatta (9.8%) were caught fire easily and sustained more burn injuries, when compared to those who were wearing clothes reaching down to the knee such as kurta, frock, skirt (15.6%) and short fitting dresses e.g. shirts, blouses, vests (9%), (*p*=0.003, Table 3). Percentage of burn was higher among wearers of synthetic fabrics (50.89%) than that of cottons (20.53%) (*p*=0.028, Table 4). 

**Table 3:** Distribution according to percentage of burns and various types of clothes worn at the time of burn

**Type of dress burnt**	**TBSA of Burns**						**Total**	
	**10- 30%**		**31-50%**		**51-70%**			
	**Male **	**Female **	**Male **	**Female **	**Male **	**Female **	**Male **	**Female **
Salwar	-	18 (8.0)	-	16 (7.14)	-	16 (7.14)	-	50 (22.3)
Dupatta	-	8 (3.5)	-	10 (4.4)	-	4 (1.7)	-	22 (9.8)
Sari	-	22 (9.8)	-	24 (10.7)	-	46 (20.5)	-	92 (41.1)
Maxi	10 (4.4)	-	6 (2.6)	-	2 (0.7)	-	18 (8.0)	-
Scarf/towel	4 (1.7)	-	4 (1.7)	-	2 (0.7)	-	10 (4.4)	-
Lungi	6 (2.6)	-	2 (0.7)	-	2 (0.7)	-	10 (4.4)	-
Dhoti	4 (1.7)	-	2 (0.7)	-	2 (0.7)	-	8 (3.5)	-
Middi/gown	4 (1.7)	-	0	-	0	-	4 (1.7)	-
Shirt	2 (0.7)	-	0	-	0	-	2 (0.7)	-
Pyjama	2 (0.7)	-	2 (0.7)	-	0	-	4 (1.7)	-
Trousers	2 (0.7)	-	2 (0.7)	-	0	-	4 (1.7)	-
Total	34 15.17)	48 (21.4)	16 (7.14)	50 22.3)	8 (3.5)	66 (29.4)	58 (25.8)	164 (73.2)

TBSA: Total body surface area

**Table 4:** Distribution according to percentage of burns and material of clothing worn

**Fabric of dress**	**TBSA of burns**			**Total**
	**10-30%**	**31-50%**	**51-70%**	
Synthetic	12 (5.3)	26 (11.6)	76 (33.9)	114 (50.89)
Semi-synthetic	4 (1.78)	34 (15.17)	26 (11.6)	64 (28.57)
Cotton	4 (1.78)	30 (13.9)	12 (5.3)	46 (20.53)
Total	20 (8.9)	90 (40.17)	114 (50.89)	224 (100)

(Chi square test) X^2^=11.466, df=2, *p*=0.028, TBSA: Total body surface area

## DISCUSSION

Burn injuries have been a major cause of concern since prehistoric days to the present era of modern medicine. Burn injuries continue to cause morbidity and mortality internationally.^12^^-^^14^ Despite international collaborations and preventative measures, there are still many cases reported in high and low-income countries. As per the available reports from India^15^^,^^16^ and other developing countries that flame (80.1%) was the most common cause of burn, although equipment responsible varied widely.^17^

In our study, large number of burn cases was due to the flame and the majority of the victims were females (59.3%). According to Feller *et al.*’s study*,* the burn patients with clothing ignition had a fourfold increase in mortality and a prolonged hospital stay when compared to those patients whose clothing was not burned.^10^ Long, loose, flowing garments like sarees, salwar, dupatta easily reached out to the fire due to unsafe cooking appliances and burning on the floor in an overcrowded kitchen. In our study, the garments such as sarees (41.1%), salwar (22.3%), and dupatta (9.8%) were caught fire easily in comparison to those who were wearing clothes reaching down to the knee such as kurta, frock, skirt (15.6%) and short fitting dresses (9%), (*p*=0.003). 

Percentage of total body surface area associated with clothing items reported by Laughlin *et al.* where in it is pointed out that the burnt area was less than 10% of the body surface in case of the close fitting garments, whereas it was greater than 10% in case of loose and flowing garments.^18^ Belshaw and Jerram also subjectively classified the garments involved in burns accidents, as the free flowing garments such as nightdress and dressing gowns to have higher risk of fatalities when compared to tight fitting garments.^19^

In our findings with respect to involvement of garment in clothing-related fire incidences revealed the same finding that the clothing material such as synthetic (50.89%) was involved in far more accidents than semi-synthetic (28.57%), and cotton (20.53%) (*p*=0.028), because synthetic fabrics are cheap and easy to maintain in comparison to cotton; but burn faster and inflict more extensive bums. This explains higher mortality among the victims who wore synthetic fabric. Patients with age between 21-40 years were more involved in burn injuries (55.8%), while females had ahigh incidence rate (31.25%). 

Bawa Bhalla *et al.* in their study regarding burn characteristics of fabric used in India reported that loose fittings garments burned vigorously and with large flames, whereas tight fitting garments were difficult to burn.^20^ Another study by Robinson demonstrated that in serious burns sustained from wearing saree, severe burns occurred due to ignition of loose fitting clothing; while saree in Asian countries was more fatal. Dense fabrics such as ‘cotton,’ burn slower.^21^ Loose fitting garments with thin fabrics such as saree were found to be much more hazardous than tight fitting garments with thick fabric, such as jeans with shirt and ‘khadi’ kurta with pajamas.^22^ In the present study, TBSA burn was extensive (50.89%) in clothing garment with synthetic material when compared to semisynthetic (28.57%) and cotton (20.53%) respectively (*p*=0.028). 

In this study, we conducted a comprehensive study on comparison of various garments material and its properties in women’s dress assemblies (saree, salwar-khameez and nightgown) and men’s dress assemblies (kurta-pyjama, shirt-pant and lungi), at the time of burn injury. Main reasons of victims were inability to identify fabric and lack of knowledge and inability to note the difference. Consumers considered only color, new design and material, while selecting textiles and apparel. Problems faced in purchase of textiles and apparel items were attractive appearance without quality, misleading discount offers and inability to assess the quality. These injuries are preventable through design and promotion of more aggressive prevention programs especially for flame injuries occurring in the home environment through advertisements, newspapers and magazines.

## CONFLICT OF INTEREST

The authors declare no conflict of interest.
